# Influence of the Relative Displacements and the Minimum Chip Thickness on the Surface Texture in Shoulder Milling

**DOI:** 10.3390/ma16247661

**Published:** 2023-12-15

**Authors:** Lukasz Nowakowski, Slawomir Blasiak, Michal Skrzyniarz

**Affiliations:** Department of Machine Design and Manufacturing Engineering, Kielce University of Technology, al. Tysiaclecia Panstwa Polskiego 7, 25-314 Kielce, Poland; lukasn@tu.kielce.pl (L.N.); sblasiak@tu.kielce.pl (S.B.)

**Keywords:** milling process, relative displacements, surface roughness, shoulder milling, minimum chip thickness, surface roughness, tool geometry

## Abstract

The formation of surface texture in milling is a complex process affected by numerous factors. This paper focuses on the surface roughness of X37CrMoV51 steel machined by shoulder milling. The aim of the study was to develop a mathematical model to predict the surface roughness parameter Ra. The proposed model for predicting the surface roughness parameter Ra in shoulder milling takes into account the feed per tooth, f_z_, the corner radius, r_ε_, and the actual number of inserts involved in the material removal process as well as h_min_ and D(ξ). The correlation coefficient between the theoretical and experimental data was high (0.96). The milling tests were carried out on a three-axis vertical milling machine using a square shoulder face mill. The geometric analysis of the face mill shows that at a feed rate of 0.04 mm/tooth, cutting was performed by three out of five inserts, and when the feed rate exceeded 0.12 mm/tooth, material was removed by all inserts. The minimum chip thickness parameter and the standard deviation of the relative displacement increased as the feed increased. Over the whole range of feeds per tooth, the displacement increased by 0.63 µm. Higher cutting speeds resulted in lower minimum chip thicknesses and the average standard deviation of the relative displacements for the whole range of cutting speeds was 2 μm.

## 1. Introduction

The manufacturing industry is facing higher and higher requirements for the quality of machine parts produced by machining. For economic reasons, it has become essential to reduce the number of finishing operations. Much of the research in this area aims to find some convenient and efficient solutions to these problems. Shoulder milling, which combines peripheral milling with face milling, may provide an answer to these expectations. These days, it is one of the most popular milling methods as it is suitable for both roughing and finishing cuts. Another approach to surface quality enhancement in machining is to use new materials for cutting tools or apply special coatings in order to improve the cutting conditions and, consequently, the process efficiency, and, ultimately, the surface quality. Changes to the tools affect the other factors responsible for the cutting process, especially the relative displacements, i.e., the displacements in the tool and workpiece system, the magnitude and distribution of cutting forces, the tool and workpiece temperatures, the friction in the cutting zone, or the minimum chip thickness. Since some of these factors largely influence the surface quality of the finished product, many studies devoted to milling or turning use them to model the surface roughness parameters, particularly Ra and Rt.

Research into the surface texture formation takes into account the contribution of various factors. For instance, a permanent change in the chip cross-section may be due to some axial and/or radial runout of the inserts employed, with this possibly resulting from insufficient geometrical or dimensional accuracy of the cutter body. The effect of the axial runout of inserts is considered in [1]; therein, the theoretical values of the surface roughness are modeled and compared with the experimental ones. A similar problem is described in [2]; the analysis concerns the influence of the tool runout on the surface quality in dynamic milling. The modeling and simulation of the surface topography in peripheral milling based on the radial and axial runout of inserts are discussed in [3]. Differences in the performance of the inserts employed resulting from their axial and radial runouts are responsible for the occurrence of displacements in the tool–workpiece system. Vibration is not a desirable phenomenon in machining because it has a negative effect on the dimensional and geometrical accuracy of the workpiece and the durability of tools and machine tool systems. The displacements in the tool–workpiece system are responsible for lower machine performance and process efficiency. It is, thus, essential to use lower values of the cutting parameters (especially the cutting speed and feed rate), which makes the process take longer. There have been many studies on the influence of vibration on the cutting process. The effects of vibration on the surface roughness parameter Ra are discussed, for example, in [4]; the research focused on the relationship between the corner radius and the relative displacements. In a further study [5], a model is proposed to predict surface topography in milling when affected by tool vibration. The method can be used to develop a formula to optimize the surface roughness parameters. Wu et al. [6] propose the use of the cutting parameters and signal features of vibration measured in milling to predict the surface roughness of S45C steel. Many studies involved using artificial neural networks or machine learning to predict the roughness parameters. For example, in [7], a model based on an artificial neural network was employed to determine surface roughness parameters. The use of machine learning methods to predict surface roughness parameters in milling was discussed in [8]. Another approach is proposed in [9], where the Long Short-Term Memory (LSTM) method was applied to predict surface roughness in milling for S45C steel. Many studies deal with the prediction of surface roughness in machining. Yan et al. [5] developed a method for predicting the surface roughness in milling based on the information about the tool vibration. The analysis of the influence of the process parameters shows that the feed per tooth has the greatest influence on surface roughness, whereas the effect of the axial depth of cut is the lowest. Many studies have aimed to determine the influence of the key machining process parameters, i.e., the feed rate, the cutting speed, and the depth of cut, on the roughness of milled surfaces [10]. Kao et al. [11] indicate that in milling, surface roughness can be predicted from the cutting forces. Their research focused on determining the relationship between the cutting force and the surface roughness of the workpiece machined on a small-size machine tool. The analysis involved employing machine learning algorithms, multi-dimensional linear regression, and a generalized neural regression network to determine the relationship between the cutting forces and surface roughness. In the machining of free-form surfaces, the major parameter responsible for surface roughness is the shape of the machining path. These days, CAM systems are used to design the shape of the tool path. Research in this area [12] reveals that tool path strategies greatly affect the actual milling time and the workpiece surface roughness. Uzun et al. [13] analyze the influence of the four most popular machining methods available in CAM systems. In micromachining, the minimum chip thickness is one of the most important and frequently considered process parameters affecting the surface roughness parameters. There have been many studies aimed to predict the minimum chip thickness by using the stagnation point [14], or to determine it through experiments [15]. Another factor responsible for the surface roughness of the workpiece is the cutting tool. Surface roughness is dependent on the tool material as well as its micro- and macrogeometry. Different materials are used for cutting tools, and the choice depends on the tool application. Recently, with the rapid development of 3D technology, additively manufactured tools are also available.

Analysis of the literature shows that much attention is given to the prediction of surface roughness parameters, particularly Ra and Rt, as these are the most popular in industry. It can be concluded that the models developed to predict surface roughness are generally used for one type of material and specific cutting conditions.

The novelty of the current article is the development of a model for predicting the surface roughness parameter Ra in shoulder milling. The parameter is determined on the basis of the following factors: the feed per tooth f_z_, the corner radius, r_ε_, the actual number of inserts used for cutting, the minimum chip thickness, h_min_, and the standard deviation of the relative displacements in the tool–workpiece system, D(ξ). The additional novelty of the present work is the description of how to use the surface profile to determine the minimum chip thickness and how to recognize the selected areas on the profile.

## 2. Materials and Methods

The material selected for the experiments was X37CrMoV5 hot work tool steel, characterized by good ductility, thermal conductivity, fracture toughness under high temperature and water cooling conditions, hardenability, and resistance to tempering. The steel is suitable for die-casting molds and cores, hot scissors and guillotine cutters, press elements, light-metal forming dies, and plastic molding cores [16]. The material has been purchased from a commercial source that certifies the chemical composition of the material. The chemical composition of X37CrMoV51 steel is provided in Table 1.

The cutting was performed using a 490-050Q22-08M milling cutter (Sandvik Coromant, Sandviken, Sweden) with five 8 mm × 3.97 mm 490–08T308M–PL–1030 inserts. The inserts, with a corner radius, r_e_, of 0.8 mm, are designed for light roughing to finishing operations. Although the maximum depth of cut for this type of insert is 5.5 mm, the producer recommends that it should not exceed 4 mm. The tool was mounted in the machine tool spindle using an A1B05–4022035 arbor (Sandvik Coromant, Sandviken, Sweden), which is an ISO 40 taper face mill arbor commonly used in machining centers.

The shoulder milling tests were carried out on a VMC 800 vertical machining center (FOP AVIA, Warsaw, Poland). The VMC 800 is a robust and stable machining center composed of four iron cast elements bolted together. The design of the VMC800 vertical machining center is based on a cross table moving in the X and Y axes and a horizontal spindle box moving in the Z axis along the vertical column. During the test, other factors in addition to the feed rates were controlled, namely, cutting speed, depth of cut, and workpiece position, and the relative displacement between tool and workpiece was measured.

A test rig was fitted on the machining center to measure the relative displacements between the tool and the workpiece; the major measuring device was an XL-80 laser interferometer (Renishaw plc, New Mills Woton-under-Edge Gloucestershire, Kingswood, UK). The measurement was performed for 20 s at a frequency of 500 Hz. A total of 10,000 measurement points were used in each measurement. The laser was mounted outside the machine to avoid vibration affecting its position. Before cutting, the tool was run through to measure any external factors that could affect the results. The displacement was then measured during cutting. The forward trend was then subtracted from the measured signal without removing any material.

Before the cutting tests, the insert mounting accuracy was measured using a Heidenhain TT120 tool touch probe (Dr. Johannes Heidenhain GmbH, Traunreut, Germany). 

The surface topography, including roughness, was analyzed using a Taylor Hobson: Talysurf CCI—Lite Non-Contact 3D profiler (Taylor-Hobson Ltd., Leicester, UK) with a 20× magnifying lens. A measurement area of 0.8 mm × 0.8 mm provided a 1024 × 1024 pixel array. The 2D surface roughness parameters were determined using a Gaussian filter with a 0.8 cut-off wavelength. 

**Figure 1:**
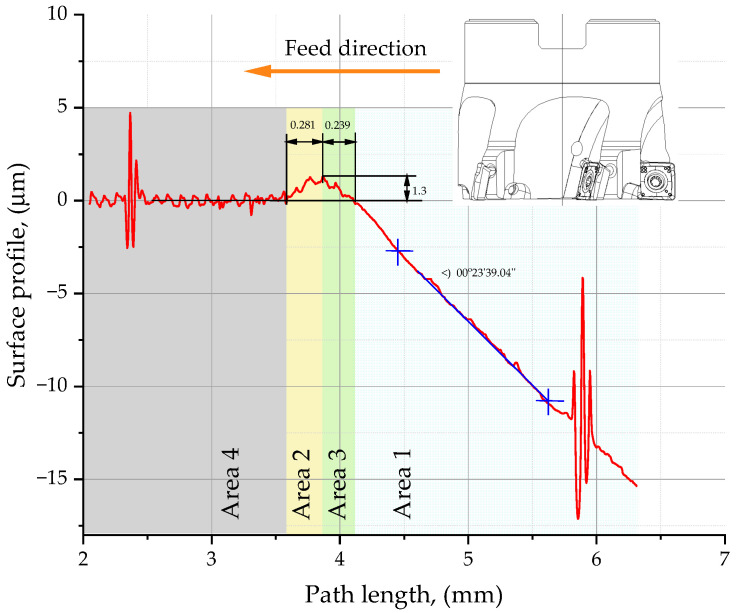
Profile used to determine the minimum chip thickness. Measurement with a TOPO 01P profilometer with a non-sliding stylus capturing features over a distance of 10 mm, in a range of 40 μm at a resolution of 1 μm.

The primary surface profile measurements for use in determining the minimum chip thickness, h_min_, were performed using a TOPO 01P profilometer (Instytut Zaawansowanych Technologii Wytwarzania, Cracow, Poland), which is a contact-type instrument equipped with a non-sliding stylus for measuring surface roughness. The profiles were analyzed using the Topografia software running on the profilometer. The profile showed two clear peaks, which corresponded to the scratches made to act as a frame of reference (a system of coordinates) for a specimen. The distance between the scratches was 3.528 mm. In Figure 1, the plot is divided into two parts, one representing the average surface profile in milling and the other the average surface profile in grinding. The angle between the lines was measured (*α* = 0°23′39″). This angle corresponded to the angle of the workpiece surface inclination to the tool during a cutting test. The profile obtained for the milled surface was smoothened in relation to the profile reported for the ground surface so that the former corresponded to zero on the axis of ordinates. The value of h_min_ (1.34 µm) was measured directly on the profile.

Because of the principle of operation of the test rig used to measure the displacements, the machining was performed in the presence of a cutting fluid. A selective static program was used to determine the effect of each factor separately. One series of the shoulder milling tests was conducted at a constant depth of cut (*a_p_* = 0.2 mm), a constant cutting speed (*v_c_* = 300 m/min), and a variable feed per tooth ranging from 0.02 to 0.22, which changed every 0.02 mm/tooth. The other series of shoulder milling tests were carried out at *a_p_* = 0.2 mm, *f_z_* = 0.1 mm/tooth, and *v_c_* = 200–400 m/min changed every 20 m/min.

## 3. Results

The axial and radial runouts of the tool head were calculated for each insert separately on the basis of 50 measurements. This required determining the average axial insert mounting accuracy. For simplicity and greater transparency, it was assumed that the tool length for the most axially protruding insert was 0 mm. Assumptions about tool engagement and zero tool length were used for calculations only to assess the radial and axial runout of the tool. A model of the cutting tool was developed to perform simulations and assess how many inserts were engaged in the surface texture generation at a given value of the feed per tooth. The model can be used to calculate the load each insert is subjected to, the actual feed per tooth, the instantaneous depth of cut, and the cross-sectional area of the material removed from each insert. Some of the calculation results are given in Table 2.

**Figure 2:**
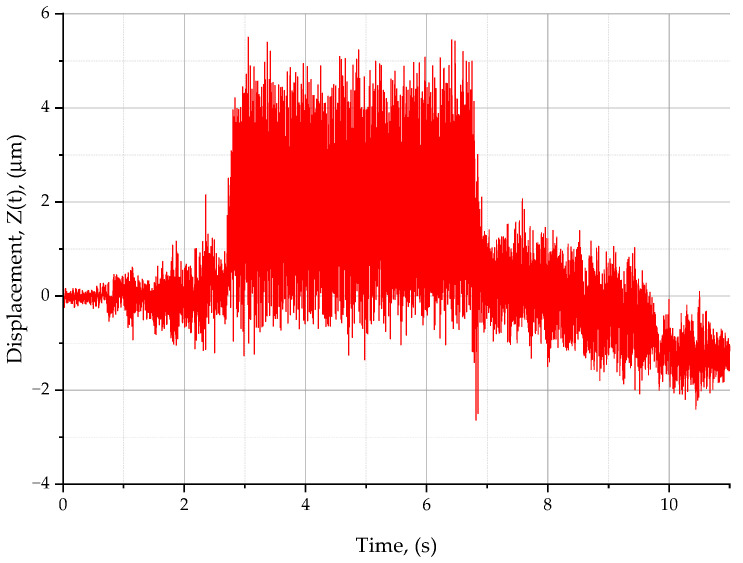
Relative displacements in the tool–workpiece system at *v_c_* = 300 m/min and *f_z_* = 0.1 mm/tooth.

From Table 2, it can be concluded that the insert mounting accuracy and the feed per tooth have a substantial influence on the material removal process. As can be seen from Table 2, only three out of five inserts were engaged in the cutting at a feed of 0.04 mm/tooth. When, however, the feed exceeded 0.12 mm/tooth, the material was removed by all the five inserts. The third insert at a feed rate of 0.12 mm/tool removes less material than the value of the h_min_ parameter, and at a feed rate of 0.14 mm/tool, the insert exceeds the value of the h_min_ parameter. During the cutting tests, the relative displacements in the tool–workpiece system were measured using an XL-80 laser interferometer (Renishaw plc, New Mills Woton-under-Edge Gloucestershire, Kingswood, UK). Some relative displacements in the tool–workpiece system are illustrated in Figure 2. 

Figure 2 shows a signal of the relative displacements registered in the tool–workpiece system during a test measurement under the following cutting conditions: *v_c_* = 300 m/min, *f_z_* = 0.1 mm/tooth, and *a_p_* = 0.2 mm. As can be seen, the signal varies over time. During the first phase, the displacement is stable because this represents the time when the tool approaches the workpiece and the tool is not engaged in cutting (0–2.7 s). In the second phase (2.7–6.8 s), when the tool performs shoulder milling, the displacement increases. The last phase (6.8–11 s) corresponds to the final stage of cutting, during which the tool is no longer in contact with the workpiece. From Figure 2, it can be concluded that the relative displacements increase with the depth of cut. Thus, it is clear that the displacements occurring in the first phase increase with increasing width of cut until the maximum engagement of the cutting tool is achieved, i.e., the whole diameter of the tool is used. It was then vital to determine how the cutting speed, v_c_, and the feed per tooth, f_z_, affected the standard deviation of the relative displacements (D(ξ)) for the selected material. Table 3 shows the standard deviation of the relative displacements measured in the tool–workpiece system, D(ξ), given in μm, for the cutting performed at *v_c_* = 300 m/min, *a_p_* = 0.2 mm, and a variable feed per tooth. 

Table 3 shows the values of the standard deviation of the relative displacement in the tool–workpiece system, i.e., the parameter D(ξ) expressed in μm, for the selected workpiece material machined at a constant cutting speed, v_c_, of 300 m/min and a variable feed per tooth, and also at a constant feed of 0.1 mm/tooth and a variable cutting speed. From the data in Table 3, it is clear that increasing the feed per tooth had a negative effect on the D(ξ) parameter. The higher the feed per tooth, the higher the standard deviation of the relative displacement. In the entire range of feeds per tooth, the displacement rose by 0.63 µm. When the feed varied between 0.02 mm/tooth and 0.08 mm/tooth, the parameter D(ξ) first increased from 1.07 µm to 1.53 µm and then dropped slightly to 1.49 µm. At feeds of 0.1–0.16 mm/tooth, D(ξ) improved from 1.49 µm to 1.61 µm to fall slightly at a feed of 0.16 mm/tooth. When the feed was 0.2 mm/tooth, there was a rapid decline in the standard deviation of the displacement to 1.48 µm, followed by a significant increase of 0.23 µm.

At feed rates of 0.10 mm/tooth and 0.16 mm/tooth, D(ξ) decreases because the third and fourth inserts were involved in surface texture generation and the minimum feed rate was higher than the h_min_ parameter. 

Two characteristic maximum values of the standard deviation of the relative displacements (2.6 μm and 3.45 µm) can be observed in Table 3. They were registered at cutting speeds of 260 m/min and 360 m/min, respectively. The points correspond to the two most efficient ranges of operation of the CoroMill 490 shoulder mill taking into consideration the displacements in the tool–workpiece system. The smallest displacements were reported at cutting speeds of 200–220 m/min, 280–340 m/min, and 400 m/min. The average standard deviation of the relative displacements for the whole range of cutting speeds was 2 μm. 

The experiment consisted of machining 22 samples, 11 at constant feed and 11 at constant cutting speed. Table 3 shows the values of the standard deviation of the relative displacement in the tool–workpiece system for the whole range of the cutting path. Discrepancies in results for the same machining parameters may be the result of material heterogeneity. The difference in the standard deviation of relative displacements for the same machining parameters is 0.39 µm. 

The next series of tests aimed to determine the influence of the cutting conditions on the minimum chip thickness (h_min_) in shoulder milling. Table 4 shows the values of the minimum cut thickness measured at different feeds per tooth and cutting speeds. 

Table 4 shows the influence of cutting speed and feed rate on the minimum chip thickness, h_min_. From the data, it can be seen that in the initial phase, an increase in cutting speed (*v_c_* = 200–260 m/min) contributed to a decrease in h_min_. The parameter h_min_ increased when the cutting speed exceeded 280 m/min. The downward trend continued until the cutting speed reached 380 m/min. The parameter remained stable until v_c_ was 260 m/min. When the cutting speed exceeded this value, h_min_ increased and then decreased. The analysis of the influence of the feed per tooth, f_z_, on the minimum chip thickness, h_min_, for X37CrMoV51 steel shows that the parameter h_min_ fluctuates up and down. From the relationship between f_z_ and h_min_, it can be concluded that the parameter h_min_ increases as the feed per tooth increases. 

**Figure 3:**
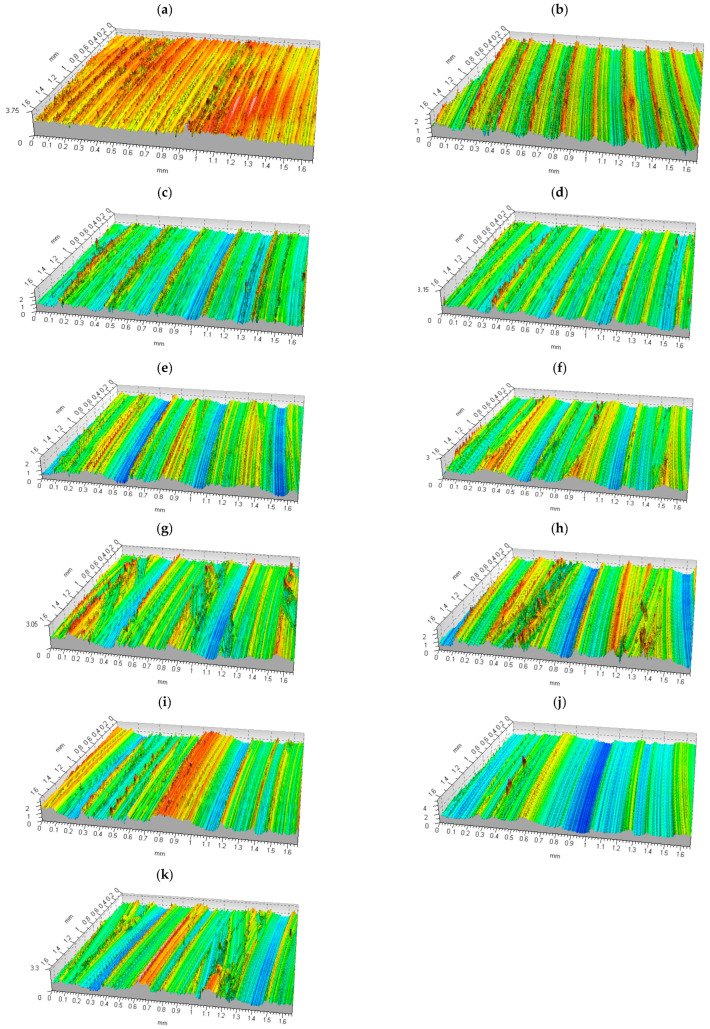
Isometric views of surfaces of the X37CrMoV51 steel specimens machined at different feeds per tooth. (**a**) *f_z_* = 0.02 mm/tooth, *f_n_* = 0.1 mm/rev, *f_t_* = 191 mm/min; (**b**) *f_z_* = 0.04 mm/tooth, *f_n_* = 0.2 mm/rev, *f_t_* = 382 mm/min; (**c**) *f_z_* = 0.06 mm/tooth, *f_n_* = 0.3 mm/rev, *f_t_* = 573 mm/min; (**d**) *f_z_* = 0.08 mm/tooth, *f_n_* = 0.4 mm/rev, *f_t_* = 764 mm/min; (**e**) *f_z_* = 0.1 mm/tooth, *f_n_* = 0.5 mm/rev, *f_t_* = 955 mm/min; (**f**) *f_z_* = 0.12 mm/tooth, *f_n_* = 0.6 mm/rev, *f_t_* = 1146 mm/min; (**g**) *f_z_* = 0.14 mm/tooth, *f_n_* = 0.7 mm/rev, *f_t_* = 1338 mm/min; (**h**) *f_z_* = 0.16 mm/tooth, *f_n_* = 0.8 mm/rev, *f_t_* = 1529 mm/min; (**i**) *f_z_* = 0.18 mm/tooth, *f_n_* = 0.9 mm/rev, *f_t_* = 1720 mm/min; (**j**) *f_z_* = 0.2 mm/tooth, *f_n_* = 0.1 mm/rev, *f_t_* = 1911 mm/min; (**k**) *f_z_* = 0.22 mm/tooth, *f_n_* = 1.1 mm/rev, *f_t_* = 2102 mm/min.

Another objective of this study was to look at the influence of the cutting process parameters in shoulder milling on the surface texture of the workpiece. The shoulder milling tests were carried out on an AVIA VMC800 vertical machining center (FOP AVIA, Warsaw, Poland). First, the tests were conducted under dry cutting conditions, i.e., without the use of cutting fluid, under specific cutting conditions assumed for a given series. The measurement results obtained for different cutting parameters were analyzed thoroughly. The data in the form of tables, plots, and maps were used to determine the 2D surface roughness parameters. Some isometric views of the surfaces of the X37CrMoV51 steel specimens are shown in Figure 3. The surface topography was analyzed using a Taylor Hobson: Talysurf CCI—Lite Non-Contact 3D profiler measurement interferometer (Taylor-Hobson Ltd., Leicester, UK).

As can be seen from Figure 3, at a feed of 0.02 mm/tooth, random roughness was observed. The surface roughness increased with increasing feed per tooth; the peak-to-valley distance corresponded to the distance the tool traveled during a single spindle rotation (feed per revolution). At *f_z_* = 0.06 mm/tooth, there were no sharp peaks; burrs were observed instead. A further increase in the feed per tooth caused an increase in both the surface roughness and the surface waviness. From the isometric views, it is evident that at *f_z_* = 0.1–0.16 mm/tooth and 0.22 mm/tooth, a two-directional cut was reported because the milling cutter milled forward and backward.

**Figure 4:**
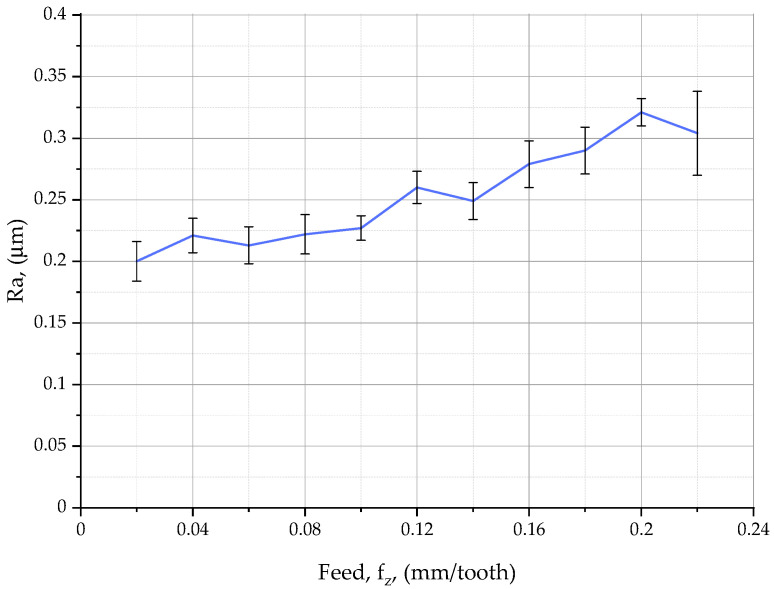
Relationship between the feed per tooth and the surface roughness parameter Ra in the shoulder milling of X37CrMoV51 steel.

Table 5 shows the 2D surface roughness parameters for shoulder milling. The lowest value of the maximum profile height, Rz, was recorded at a feed rate of 0.1 mm/tooth, while the highest value was recorded at a feed rate of 0.22 mm/tooth. The profile characteristics, Rt and Rv, were similar to Rz. The arithmetic mean height, Ra, (Figure 4) increased steadily with increasing feed from 0.2 μm at 0.02 mm/tooth to 0.321 μm at 0.2 mm/tooth. At a feed of 0.22 mm/tooth, Ra decreased slightly to 0.304 μm. The root mean square deviation, Rq, and Rp, the maximum profile peak height, were reported to be identical to Ra over the whole range of feeds. The mean height of the profile elements, Rc, decreased with increasing feed. After the feed exceeded 0.06 mm/tooth, Rc fluctuated around 0.55 μm and then increased when the feed was 0.1–0.18 mm/tooth. At feed rates higher than 0.18 mm/tooth, the parameter Rc decreased. The mean width of the profile elements, RSm, first increased and then decreased with increasing feed per tooth. At *f_z_* = 0.06 mm/tooth, RSm decreased to 0.03 mm. Then, at feed rates of 0.08–0.12 mm/tooth, RSm increased to 0.048 mm and then decreased to 0.034 mm. A further increase in feed to 0.2 mm/tooth caused the parameter RSm to reach a maximum of 0.058 mm and then drop to 0.04 mm. 

The next stage of the study was to develop a model for predicting the surface roughness parameter, Ra, in shoulder milling. First, relationship (1) was formulated as a linear function to link the minimum chip thickness with the feed per tooth. The coefficients used in the formula were selected in such a way that the feed could be expressed in mm. As there were substantial fluctuations in h_min_, the model took into consideration only the minimum chip thickness, which increased with increasing feed per tooth.

(1) $$h_{min}=2.6536{\cdot f}_{z}+0.58$$

Then, relationship (2) was formulated in the form of a power function to calculate the parameter D(ξ) using the value of the feed per tooth. The coefficient of correlation between the theoretical results determined from relationship (2) and the experimental data obtained through tests was 0.89.

(2) $$D\left(\xi \right)=2.1461\cdot {f_{z}}^{0.1636}$$

The last stage of the study was to formulate relationship (3), which is the model for predicting the surface roughness parameter, Ra, on the basis of the number of inserts engaged, the corner radius, the minimum chip thickness, and the relative displacements. It was assumed that the relative displacements combined with the geometry of the insert (wiper for finishing) contribute to lower roughness as they help remove some of the machining debris.

(3) $$Ra=\frac{{f_{za}}^{2}}{8\cdot r_{\varepsilon }}+\frac{{k\cdot h}_{min}}{D(\xi )}$$

where f_za_ is the actual value of the feed per tooth dependent on the actual number of inserts taking part in the cutting process, z_a_.

(4) $$f_{za}=\frac{f_{z}\cdot z}{z_{a}}$$

k is the material coefficient describing the contribution of the minimum chip thickness to the surface roughness formation. The material coefficient, k, is the ratio of the average peak-to-valley distance on a surface profile to the minimum chip thickness.

(5) $$k=\frac{\left(\frac{\sum_{i=1}^{n}a_{n}}{n}\right)}{h_{min}}=\frac{\left(\frac{0.30+0.25+0.29+0.29}{4}\right)}{0.75}=0.373$$

The analysis to determine the effect of the selected factors required finding the value of the material coefficient, k. The average value of this coefficient (*k* = 0.4) was calculated using the data from measurements of 11 X37CrMoV51 steel specimens.

The model for predicting the surface roughness parameter, Ra, in shoulder milling was verified using relationship (3) by substituting the actual feed per tooth with relationship (4), the material coefficient, k, with relationship (5), and the parameters h_min_ and D(ξ) with relationships (1) and (2), respectively. The predicted surface roughness Ra was thus dependent on the feed per tooth, f_z_, the corner radius, r_ε_, the actual number of inserts taking place in the material removal process, and the parameters h_min_ and D(ξ).

Table 6 shows the data used to verify the proposed model. As can be seen, the difference between the measured values and the calculated values is very small.

**Figure 5:**
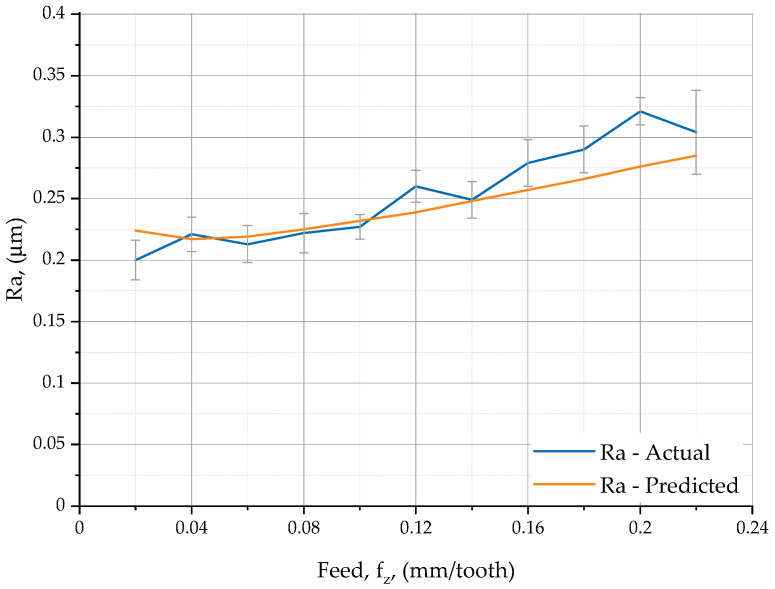
Actual and predicted surface roughness of face milled X37CrMoV51steel versus feed per tooth.

Figure 5 compares the plots obtained from the experiments with those from the calculations.

The red line in Figure 5 represents the predicted values of the surface roughness parameter Ra in shoulder milling, which were calculated using the proposed model based on relationship (3). The blue line shows the relationship between the actual surface roughness and the feed per tooth in shoulder milling at a cutting speed of 300 m/min and a depth of cut of 0.2 mm. The X37CrMoV51 steel specimens were measured with a Taylor Hobson Talysurf CCI–Lite Non-Contact 3D Profiler (Taylor-Hobson Ltd., Leicester, UK). 

From Table 6 and Figure 5, it is evident that for feed rates ranging from 0.02 to 0.18 mm/tooth, the predicted surface roughness Ra determined using Equation (3) is similar to the actual surface roughness. At a feed rate of 0.2 mm/tooth, however, the predicted values are slightly different from the actual ones. The coefficient of correlation between the theoretical and experimental data was 0.96. This value confirms that the developed model is correct.

## 4. Conclusions

The major conclusions drawn from the shoulder milling experiments for X37CrMoV51 steel are as follows:

1. The material removal process was largely affected by the feed per tooth and the number of inserts engaged in the cutting process. The analysis of the experimental data reveals that at a feed of 0.04 mm/tooth, the cutting was performed by three out of five inserts. However, when the feed exceeded 0.12 mm/tooth, the material was removed by all the inserts. At feed rates of 0.10 mm/tooth and 0.16 mm/tooth, D(ξ) decreases because the third and fourth inserts were involved in surface texturing and the minimum feed rate was higher than the value of the h_min_ parameter.
2. The average standard deviation of the displacement in the tool–workpiece system for the whole range of cutting speeds was 2 μm. The smallest relative displacements were recorded at cutting speeds of 200–220 m/min, 280–340 m/min, and 400 m/min, so these parameters guarantee the most stable conditions.
3. Higher cutting speeds resulted in lower minimum chip thicknesses, h_min_. However, the parameter h_min_ increased with increasing feed. Under variable feed conditions, the lowest and highest values of h_min_ were 0.276 μm and 1.71 μm, respectively. Under variable cutting speed conditions, however, the lowest value of h_min_ was 0.277 μm, while the highest was 1.48 μm.
4. The parameter Ra showed a gradual increase over the entire range of feed rates: from a minimum of 0.2 μm at a feed of 0.02 mm/tooth to a maximum of 0.321 μm at 0.2 mm/tooth. At *f_z_* = 0.1–0.16 mm/tooth and 0.22 mm/tooth, a two-directional cut was reported because the milling cutter milled forward and backward.
5. The most favorable machining conditions, based on the lowest values of h_min_, standard deviation of the displacement, and the number of inserts involved in the cutting process, is a feed rate of 0.10 mm/tooth.

## Figures and Tables

**Table 1:** Composition of the alloying elements of the workpiece material, %.

C	Mn	Si	P	S	Cr	Ni	Mo	W	V	Co	Cu
0.32–0.42	0.2–0.5	0.8–1.2	max. 0.03	max. 0.03	4.5–5.5	max. 0.35	1.2–1.5	max. 0.3	0.3–0.5	max. 0.3	max. 0.3

**Table 2:** Theoretical versus actual feed per tooth for each insert.

Theoretical Feed, fz, mm/tooth	Actual Feed in mm/tooth for Each Insert				
	1	2	3	4	5
0.02	0.011	0.005	0	0.004	0
0.04	0.019	0.009	0	0.012	0
0.06	0.027	0.013	0	0.019	0.001
0.08	0.035	0.017	0	0.027	0.001
0.1	0.043	0.021	0	0.034	0.002
0.12	0.048	0.025	0.001	0.041	0.005
0.14	0.052	0.029	0.003	0.046	0.01
0.16	0.056	0.033	0.007	0.05	0.014
0.18	0.061	0.037	0.01	0.054	0.018
0.2	0.065	0.041	0.014	0.058	0.022
0.22	0.069	0.045	0.017	0.062	0.027

**Table 3:** Variation in the parameter D(ξ) with feed rate and cutting speed.

*v_c_* = 300 m/min *a_p_* = 0.2 mm		*f_z_* = 0.1 mm/tooth *a_p_* = 0.2 mm	
f_z_, mm/tooth	D(ξ), μm	v_c_, m/min	D(ξ), μm
0.02	1.07	200	1.75
0.04	1.29	220	1.75
0.06	1.36	240	2.10
0.08	1.53	260	2.6
0.1	1.49	280	1.86
0.12	1.56	300	1.88
0.14	1.61	320	2.03
0.16	1.56	340	1.97
0.18	1.61	360	3.45
0.2	1.48	380	2.04
0.22	1.71	400	1.48

**Table 4:** Variation in the values of h_min_ with feed per tooth, f_z_, and cutting speed, v_c_.

*v_c_* = 300 m/min *a_p_* = 0.2 mm		*f_z_* = 0.1 mm/tooth *a_p_* = 0.2 mm	
f_z_, mm/tooth	h_min_, μm	v_c_, m/min	h_min_, μm
0.02	0.706	200	0.8
0.04	0.65	220	0.695
0.06	0.276	240	0.674
0.08	1.113	260	0.465
0.1	0.95	280	0.949
0.12	0.657	300	0.42
0.14	1.312	320	0.508
0.16	1.047	340	0.431
0.18	1.313	360	0.277
0.2	0.713	380	1.07
0.22	1.71	400	1.48

**Table 5:** Variation in the values of the 2D surface roughness parameters in the shoulder milling of X37CrMoV51 steel with the feed per tooth.

Feed per Tooth f_z_, mm/tooth												
Parameter	Unit	0.02	0.04	0.06	0.08	0.1	0.12	0.14	0.16	0.18	0.2	0.22
Rp	µm	0.961	0.981	1.106	1.135	1.032	0.992	1.164	1.202	1.004	1.225	1.376
Rv	µm	1.112	0.759	0.691	0.801	0.661	0.768	0.784	0.891	0.847	0.869	0.970
Rz	µm	2.074	1.740	1.797	1.936	1.693	1.760	1.948	2.093	1.851	2.095	2.346
Rc	µm	0.646	0.620	0.509	0.521	0.530	0.685	0.675	0.774	0.809	0.751	0.717
Rt	µm	2.078	1.748	1.798	1.952	1.696	1.782	2.035	2.114	1.915	2.111	2.352
Ra	µm	0.200	0.221	0.213	0.222	0.227	0.260	0.249	0.279	0.290	0.321	0.304
Rq	µm	0.272	0.279	0.278	0.285	0.286	0.314	0.315	0.348	0.349	0.402	0.384
RSm	mm	0.024	0.032	0.030	0.030	0.036	0.048	0.034	0.043	0.050	0.058	0.040

**Table 6:** Data used to verify the proposed model.

X37CrMoV51 Steel	Actual Number of Inserts	Predicted Roughness	Actual Roughness	Standard Deviation
Feed, f_z_, mm/tooth	z_rz_, Number	Ra, μm	Ra, μm	σ, μm
0.02	3	0.224	0.2	0.016
0.04	3	0.217	0.221	0.014
0.06	4	0.219	0.213	0.015
0.08	4	0.225	0.222	0.016
0.1	4	0.232	0.227	0.01
0.12	5	0.239	0.26	0.013
0.14	5	0.248	0.249	0.015
0.16	5	0.257	0.279	0.019
0.18	5	0.266	0.29	0.019
0.2	5	0.276	0.321	0.011
0.22	5	0.285	0.304	0.034
		Coefficient of correlation *R*^2^ = 0.96		

## Data Availability

The data presented in this study are available on request from the corresponding author.
